# Transporter Proteins as Therapeutic Drug Targets—With a Focus on SGLT2 Inhibitors

**DOI:** 10.3390/ijms25136926

**Published:** 2024-06-25

**Authors:** Nina Komaniecka, Sonia Maroszek, Maria Drozdzik, Stefan Oswald, Marek Drozdzik

**Affiliations:** 1 Department of Experimental and Clinical Pharmacology, Pomeranian Medical University, 70-111 Szczecin, Poland; nina.komaniecka@pum.edu.pl (N.K.); sonia.maroszek@pum.edu.pl (S.M.); mariadrozdzik@gmail.com (M.D.); 2 Institute of Pharmacology and Toxicology, Rostock University Medical Center, 18057 Rostock, Germany; stefan.oswald@med.uni-rostock.de

**Keywords:** targeted therapy, flozins, myrcludex B, odevixibat, maralixibat, probenecid, NTCP, ASBT, OAT1, OAT3, URAT1

## Abstract

Membrane transporters interact not only with endogenous substrates but are also engaged in the transport of xenobiotics, including drugs. While the coordinated function of uptake (solute carrier family—SLC and SLCO) and efflux (ATP-binding cassette family—ABC, multidrug and toxic compound extrusion family—MATE) transporter system allows vectorial drug transport, efflux carriers alone achieve barrier functions. The modulation of transport functions was proved to be effective in the treatment strategies of various pathological states. Sodium–glucose cotransporter-2 (SGLT2) inhibitors are the drugs most widely applied in clinical practice, especially in the treatment of diabetes mellitus and heart failure. Sodium taurocholate co-transporting polypeptide (NTCP) serves as virus particles (HBV/HDV) carrier, and inhibition of its function is applied in the treatment of hepatitis B and hepatitis D by myrcludex B. Inherited cholestatic diseases, such as Alagille syndrome (ALGS) and progressive familial intrahepatic cholestasis (PFIC) can be treated by odevixibat and maralixibat, which inhibit activity of apical sodium-dependent bile salt transporter (ASBT). Probenecid can be considered to increase uric acid excretion in the urine mainly via the inhibition of urate transporter 1 (URAT1), and due to pharmacokinetic interactions involving organic anion transporters 1 and 3 (OAT1 and OAT3), it modifies renal excretion of penicillins or ciprofloxacin as well as nephrotoxicity of cidofovir. This review discusses clinically approved drugs that affect membrane/drug transporter function.

## 1. Introduction

The cellular disposition of various endogenous compounds and xenobiotics depends in part on transport proteins located in the cell membrane. They are usually divided into two main superfamilies: the ATP-binding cassette (ABC) family and the solute carrier (SLC) family. Until now, over 500 human transport proteins have been described and characterized. Forty-nine members of ABC proteins are primary active transporters that use the energy of ATP hydrolysis to efflux substrates across the membrane and are classified on the basis of their abundance into seven subfamilies (A–G) [1,2]. One characteristic shared by ABC transporters is the presence of two nucleotide-binding domains (NBDs) and two transmembrane domains (TMDs), which identify the substrate and facilitate its transport across the cell membrane [3]. Certain ABC genes are known to encode proteins that are classified as half-transporters, which are composed of one transmembrane domain and one nucleotide-attaching domain, such as the breast cancer resistance protein—BCRP (ABCG2) [4]. More than 450 SLC proteins exhibit amino acid sequence similarities ranging from 25–30% and are grouped into 66 families, ranging between 1 and 53 genes/proteins per family [5]. Unlike ABC transporters, SLC transporters lack ATP-binding sites [6] (pp. 1–10). SLC carriers can mediate uniport down a concentration gradient as well as secondary active transport using the symport or antiport of two substrates against a concentration gradient, regulating the efflux and influx of particular substrates [1].

ABC and SLC transporters can also be classified according to their efflux and influx functions. The list of major efflux transporters includes the following members: multidrug resistance family (MDR and ABCB), multidrug resistance-associated protein family (MRP and ABCC), breast cancer resistance protein (BCRP and ABCG2), multidrug and toxin compound extrusion family (MATE and SLC47A), and major influx carriers include: organic-anion-transporting polypeptide family (OATP and SLC21/SLCO), organic anion transporter family (OAT and SLC22A), organic cation transporters family (OCT and SLC22A) organic cation/carnitine transporter family (OCTN and SLC22A) [1].

Drug transporter proteins are distributed across body tissues and organs and mediate coordinated transport across cells or barriers. The influx carriers in the apical membrane shuttle substrates inside cells, whereas efflux transport systems eliminate a substrate or its metabolites mostly via the basolateral membrane [1]. Transport proteins that mediate the absorption and distribution of drugs may be called drug transporters, although this is not their unique function [6] (pp. 1–10). Approximately 15–20 ABC and SLC transporters/carriers have been well characterized because of their specific roles in drug transport [7]. Moreover, despite the continuous development of the field of transporter biology, we still possess insufficient knowledge of the endogenous substrates or physiological functions of more than half of the members of the transporter superfamily [2]. Obtaining atomic resolution crystal structures of membrane transporters has proven to be extremely challenging for several reasons. First, the amphipathic nature of the transporter surface presents a hydrophobic area in contact with membrane phospholipids and polar surface areas in contact with the aqueous phases on both sides of the membrane. Second, many transporters have a low abundance in the cell membrane, making them impossible to overexpress, which is a crucial requirement for structural studies. Third, the inherent conformational flexibility of transporters makes it difficult to obtain stable crystals [6] (pp. 1–10). Information about transporters/carriers substrate would allow targeted transport modulation of cell functions. The initial crucial step in developing a drug that targets membrane transporters is to comprehend the mechanisms involved in the overall transport process, as well as to identify the substrates and/or inhibitors of drug transporters [8] (pp. 65–103). However, membrane transporters/carriers are a diverse set of proteins that can transport a variety of substrates, including ions and organic molecules of varying size, and are highly flexible in structure to accommodate different substrates. This suggests that drugs may bind to different conformations of a transporter and that it is important to consider multiple conformations of a transporter when designing ligands for a targeted approach [9] (pp. 15–59). 

This review highlights transporters that serve as specific targets for drugs with a focus on structural characteristics as well as the diseases that are linked to them and delve into the manner in which drugs impact these transporters [10]. Table 1 summarizes all of the SLC transporters that are discussed in this review.

## 2. Flozins—SGLT2 Inhibitors

Sodium–glucose cotransporter SGLT2 (encoded by SGLT2, also known as SLC5A2) is a significant mediator of glucose transport in epithelial cells. In the past decade, SGLT2 inhibitors have emerged as a new mainstay in the treatment of type 2 diabetes mellitus [11]. Members of the SLC5 family code for proteins that range in size from 60 to 80 kDa and contain between 580 and 718 amino acids. This group of genes is now referred to as the sodium solute symporter family and is composed of hundreds of proteins from both prokaryotic and eukaryotic sources that share a similar structure. Proteins in this family have a consensus amino acid sequence in their structural domain [48]. 

SGLT2 is expressed in the luminal membrane in the early proximal tubule of the kidney and is responsible for all glucose reabsorption in this segment, accounting for approximately 97% of the whole kidney’s fractional glucose reabsorption (FGR) [12]. The remaining 3% of FGR is typically maintained under normal physiological conditions by a similar transporter known as sodium–glucose cotransporter SGLT1 [49].

Approximately 50 mutations in the SGLT2 gene have been linked to familial renal glucosuria, a condition that is rare and, therefore, not well studied or fully understood [11,50]. It is inherited through autosomal recessive genes. This condition causes glucose to be excreted in the urine, with daily excretion exceeding 1 g, despite normal blood glucose levels and oral glucose tolerance tests [51,52] (pp. 131–140). Individuals with SGLT2 mutations have not been observed to experience any serious complications, such as ascending urinary tract infections or impaired kidney function [48,50]. While familial renal glucosuria is generally considered a benign condition, it can cause symptoms such as polyuria, excessive thirst, nocturnal enuresis, polyphagia, and recurring urinary tract infections [11]. To date, no mutation associated with familial renal glucosuria has been tested for its functional effects on SGLT2 because of the low expression of this protein in heterologous expression systems. As a result, it is difficult to establish a direct link between the mutations and the severity of glucosuria [48].

The global economic cost of diabetes mellitus is estimated to be approximately 825 billion USD each year [51]. This condition is a major contributor to cardiovascular and end-stage renal diseases, with cardiovascular disease being the primary cause of death among patients with diabetes [53]. While there are several drug classes available for managing diabetes mellitus (such as insulin, metformin, sulfonylureas, and glitazones), they have limitations. For instance, they may not effectively improve cardiovascular outcomes, and some may even increase the risk of fatal cardiovascular events [11].

The maximum amount of glucose that the kidneys can transport from the blood is reached when blood glucose levels exceed 11.1 mmol/l, causing excess glucose to be excreted in the urine. This renal safety valve helps prevent extreme hyperglycemia. Diabetes may increase the kidneys’ maximum glucose transport capacity because of tubular growth or increased expression of SGLT2 or SGLT1 [54,55,56,57]. The enhanced glucose reabsorption in the kidneys in diabetes helps maintain hyperglycemia and is, therefore, considered maladaptive. When SGLT2 is blocked, the kidneys’ ability to reabsorb glucose decreases to the level of SGLT1, which is about 80 g per day. In other words, blocking SGLT2 causes the renal safety valve to open at a lower threshold, making it relevant to glucose homeostasis in the normal and moderately elevated glucose range [11].

Developing a drug to lower glucose levels involves the risk of hypoglycemia, which can impair cardioprotective effects due to the activation of the sympathetic nervous system [58]. The risk of hypoglycemia associated with SGLT2 inhibitors is low because of the compensation provided by SGLT1 in the late proximal tubule, which eliminates glucose excretion when filtered glucose falls below the transport capacity of SGLT1. Additionally, metabolic counter-regulatory mechanisms, such as the upregulation of hepatic gluconeogenesis, help prevent hypoglycemia [59].

**Figure 1:**
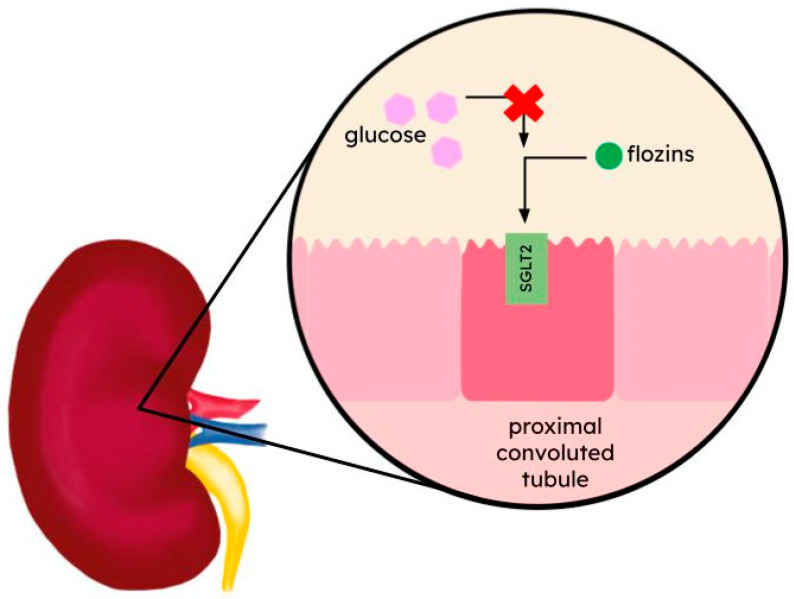
Mechanism of action of selective sodium–glucose cotransporter 2 (SGLT2) inhibitors. Flozins inhibit glucose reabsorption in the proximal renal tubule by binding with SGLT2, therefore lowering glucose serum levels.

The inhibition of glucose reabsorption in the kidneys is a promising approach to treat hyperglycemia (Figure 1). Selective SGLT2 inhibitors: dapagliflozin, canagliflozin, empagliflozin, and ertugliflozin are based on the same meta C-glycosylated diarylmethane pharmacophore. Unlike O-glucosides that have failed in previous clinical trials, C-glycosylation makes these molecules resistant to hydrolysis by β-glucosidases, resulting in increased half-life [60,61,62]. 

The initial effect of SGLT2 inhibition is a decrease in glomerular filtration rate (GFR). This reduction is caused by the increased delivery of fluid and NaCl to the distal nephron, which lowers GFR through the tubuloglomerular feedback mechanism and increases tubular back pressure [59]. This was demonstrated in streptozotocin-induced diabetic rats in 1999 using a micropuncture technique on the single nephron level and the application of the SGLT1/2 inhibitor phlorizin into the early proximal tubule [63]. It was later confirmed with dapagliflozin in 2012 [64]. By lowering GFR, albuminuria is reduced, and the oxygen-consuming transport activity in renal tubules is decreased. This helps to preserve the function and integrity of the remaining nephrons in the long term [59]. 

The use of SGLT2 inhibitors in humans [65,66,67] and the pharmacological or genetic inhibition of SGLT2 in mice [49,68] has resulted in a reduction in FGR to only around 40–50%, despite SGLT2 typically contributing to over 95% of FGR. This is because SGLT1 plays a compensatory role in the late proximal tubule [68]. Therefore, blocking both SGLT1 and SGLT2 in the kidney may be more beneficial for lowering blood glucose levels than SGLT2 inhibition alone [69]. The impact of intestinal SGLT1 inhibition on blood glucose control is expected to add to the renal effects. However, dual SGLT1/2 inhibition may increase the risk of hypoglycemia, and the stronger diuretic effect could expose patients to hypotension, pre-renal failure, haemoconcentration-related complications, and diabetic ketoacidosis [11].

### 2.1. Dapagliflozin

Dapagliflozin is an extremely potent, selective, and reversible inhibitor of SGLT2, which reduces the reabsorption of glucose from the glomerular filtrate in the proximal renal tubule. This leads to a decrease in sodium reabsorption, urinary excretion of glucose, and osmotic diuresis. Consequently, dapagliflozin increases the delivery of sodium to the distal tubule, which enhances tubuloglomerular feedback and reduces intraglomerular pressure. This results in a reduction in volume overload, blood pressure, preload, and afterload, which can have beneficial effects on cardiac remodeling, diastolic function, and preserve renal function. The drug is rapidly absorbed into the bloodstream, with peak levels achieved within 2 h of oral administration, and then declines with a half-life of 17–18 h [66]. The cardiac and renal benefits of dapagliflozin are not solely dependent on its blood glucose-lowering effect and are not limited to patients with diabetes, as demonstrated in several clinical studies [70,71,72]. Dapagliflozin also increases hemoglobin levels and reduces body weight. It improves both fasting and postprandial plasma glucose levels by reducing renal glucose reabsorption, which leads to urinary glucose excretion. This glucose excretion (glucuretic effect) is observed after the first dose, is continuous over the 24-h dosing interval, and is sustained throughout the treatment. The amount of glucose removed by the kidney through this mechanism depends on the blood glucose concentration and GFR. Therefore, in subjects with normal blood glucose levels, dapagliflozin has a low likelihood of causing hypoglycemia. Dapagliflozin does not impair normal endogenous glucose production in response to hypoglycemia and acts independently of insulin secretion and insulin action. Dapagliflozin is unique in that it does not hinder other essential glucose transporters responsible for transporting glucose into peripheral tissues. Additionally, it demonstrates over 1400 times more selectivity for SGLT2 than SGLT1, which is the primary transporter in the gut responsible for glucose absorption [73].

Dapagliflozin was registered in Europe in 2012 [74,75]. In the United States, it was registered by the U.S. Food and Drug Administration (FDA) in 2014 [76]. In 2023, a generic version of dapagliflozin was registered by the European Medicines Agency (EMA) [77]. Additionally, dapagliflozin is available in combination with metformin and saxagliptin [78,79,80].

Dapagliflozin was initially used to treat type 2 diabetes mellitus (T2DM). A randomized, double-blind, placebo-controlled, parallel-group phase 3 clinical trial was conducted across 85 centers in the USA, Canada, Mexico, and Russia between 2007 and 2010. The trial enrolled male and female participants aged 18–77 with type 2 diabetes who were inadequately controlled with diet and exercise alone. The trial consisted of a two-week diet/exercise placebo lead-in period, followed by a 24-week treatment period, during which participants were randomly assigned to one of seven groups to receive either a once-daily placebo or 2.5, 5, or 10 mg of dapagliflozin, administered either in the morning or evening. The trial included a high glycated hemoglobin (HbA1c) exploratory cohort (10.1–12% HbA1c), which was randomly assigned in a 1:1 ratio to receive treatment with either 5 or 10 mg of dapagliflozin (a placebo group was not included because of the high HbA1c levels). Treatment with dapagliflozin resulted in statistically significant reductions in HbA1c levels compared with the placebo group [13]. 

Participants who successfully completed the 24-week double-blind treatment period were eligible for a 78-week extension [81]. Following the 24-week mark, those in the placebo group were administered low-dose metformin 500 mg/day in combination with a dapagliflozin-matching placebo (placebo + low-dose metformin), whereas participants in the dapagliflozin groups continued on the same active treatments with the addition of a metformin-matching placebo. An additional exploratory cohort comprised participants with an HbA1c of 10.1–12.0%, who were randomly assigned (1:1) to receive either dapagliflozin 5 or 10 mg administered once daily in the morning. During the extension period, HbA1c reductions were sustained for up to 102 weeks. 

Dapagliflozin was well tolerated when used in combination with metformin, metformin and sulfonylurea, glimepiride, sitagliptin (with or without metformin), or insulin, and it resulted in statistically significant reductions in HbA1c at 24 weeks compared with the placebo. They were maintained in most of these add-on combination studies during the extension periods. In particular, the reductions in HbA1c observed at 24 weeks were sustained in the add-on combination studies using glimepiride and insulin for up to 48 and 104 weeks, respectively [82,83,84,85].

DURATION-8 was a phase 3, multicenter, double-blind, randomized, active-controlled trial conducted across six countries at 109 sites. A total of 685 adults aged 18 or above with T2DM and uncontrolled glycemia (HbA1c 8–12%), despite receiving stable metformin monotherapy (≥1500 mg/day) were randomly assigned in a 1:1:1 ratio to receive either once-weekly exenatide 2 mg by subcutaneous injection, once-daily dapagliflozin 10 mg oral tablets, or exenatide and dapagliflozin-matched placebos. All treatment groups showed a reduction in HbA1c levels compared with the baseline. Combination therapy with dapagliflozin 10 mg and prolonged-release exenatide was superior in reducing HbA1c levels compared with dapagliflozin alone and prolonged-release exenatide alone [86].

Subsequently, several significant clinical trials demonstrated the additional efficacy of dapagliflozin in chronic kidney disease (CKD) and heart failure. To define the cardiovascular safety of dapagliflozin, an international, multicenter, randomized, double-blind, placebo-controlled, phase 3 clinical study was conducted—Dapagliflozin Effect on Cardiovascular Events (DECLARE). The trial included patients who were at least 40 years old and had type 2 diabetes with HbA1c levels between 6.5% and 11.9%. Patients with multiple risk factors for atherosclerotic cardiovascular disease or established atherosclerotic cardiovascular disease were also eligible. During a 4-to-8-week run-in period, all patients received a placebo. Following the run-in period, 1:1 randomization was conducted in a double-blind fashion, with 17,160 participants receiving either 10 mg dapagliflozin daily or a matching placebo. A total of 6974 patients (40.6%) had established atherosclerotic cardiovascular disease, while 10,186 (59.4%) had multiple risk factors. The median follow-up period was 4.2 years. The trial demonstrated that dapagliflozin was non-inferior to the placebo in terms of the primary safety outcome of MACE (defined as cardiovascular death, myocardial infarction, or ischemic stroke). While dapagliflozin did not result in a significantly lower rate of MACE, it resulted in a significantly lower rate of cardiovascular death or hospitalization for heart failure compared with the placebo. Additionally, there were indications of a possible lower rate of adverse renal outcomes [87].

The DAPA-HF study was an international, multicenter, randomized, double-blind, placebo-controlled clinical trial conducted between 2017 and 2018 to evaluate the effect of dapagliflozin on the incidence of cardiovascular death and worsening heart failure in patients with heart failure with reduced ejection fraction. A total of 4744 patients were enrolled in the study, including 2373 patients who received dapagliflozin 10 mg and 2371 who received a placebo. The study duration was 18 months, and patients diagnosed with type 2 diabetes were randomly assigned to treatment groups. The benefits of dapagliflozin were observed early in the study and persisted throughout the duration of the trial. The results showed that dapagliflozin reduced the total number of hospitalizations for heart failure (both first and recurrent) and cardiovascular deaths. The treatment benefit of dapagliflozin was observed in heart failure patients with and without T2DM by reducing the primary composite endpoint of incidence of cardiovascular death and worsening heart failure [70]. 

The DELIVER study was an international, multicenter, randomized, double-blind, placebo-controlled trial conducted in patients aged 40 or older with chronic heart failure and a left ventricular ejection fraction of 40% or less to investigate the impact of dapagliflozin as compared with a placebo on the incidence of cardiovascular death and worsening heart failure. A total of 6263 patients were randomly assigned to receive either dapagliflozin at a dose of 10 mg once daily or a matching placebo in addition to their regular treatment. Over a median of 2.3 years, dapagliflozin showed a reduction in the combined risk of worsening heart failure or cardiovascular death among patients with heart failure and either mildly reduced or preserved ejection fraction. The total number of events and symptom burden was lower in the dapagliflozin group than in the placebo group. The results were similar in patients with a left ventricular ejection fraction of 60% or more and those with a left ventricular ejection fraction of less than 60%, as well as in prespecified subgroups, including patients with or without diabetes. The incidence of adverse events was similar in both groups [71].

The DAPA-CKD study was an international, multicenter, randomized, double-blind, placebo-controlled trial conducted to evaluate the effect of dapagliflozin on renal outcomes and cardiovascular mortality in patients with chronic kidney disease. The primary outcome was a composite of a sustained decline in estimated glomerular filtration rate (eGFR) of at least 50%, end-stage kidney disease, or death from renal or cardiovascular causes. The study included 4304 participants with an estimated GFR of 25 to 75 mL/min/1.73m^2^ of body surface area, who were randomly assigned to receive dapagliflozin (10 mg once daily) or a placebo. Over a median of 2.4 years, among patients with chronic kidney disease, regardless of the presence or absence of diabetes, the risk of a composite of a sustained decline in eGFR of at least 50%, end-stage kidney disease, or death from renal or cardiovascular causes was significantly lower with dapagliflozin than with the placebo. Treatment with dapagliflozin also led to a significant reduction in all-cause mortality and improved overall survival in chronic kidney disease patients with CKD [72].

The results of safety studies on dapagliflozin in healthy human subjects revealed that the medication is well tolerated, with only minor adverse effects and no significant changes in vital signs compared with the placebo group [66]. Common or frequently reported adverse reactions comprised genital infections, dizziness, rash, back pain, and dysuria [74]. 

### 2.2. Canagliflozin

Canagliflozin operates in a manner analogous to dapagliflozin, and at doses of 300 mg/day or higher, it may also impede SGLT1 in the intestines, thereby delaying the absorption of postprandial glucose. However, daily doses of 100 mg do not seem to exhibit a significant affinity for the SGLT1 receptor [88]. It was registered by EMA and FDA in 2013 [89,90]. It is also available in conjunction with metformin [91]. Canagliflozin achieves its highest levels in the blood within one to two hours after oral administration and reaches a steady state within four to five days. Its bioavailability is 65%, and it is largely bound to proteins, mainly albumin, with a binding rate of 99%. The half-life of the drug is approximately 10.6–13.1 h after a single oral dose. Approximately 33% of canagliflozin is eliminated through the kidneys, while around 42% is excreted in the feces [89].

CANTATA-M, a phase 3 trial spanning 26 weeks, employed a rigorous methodology featuring randomization, double-blinding, and placebo control. It aimed to evaluate the safety and efficacy of canagliflozin monotherapy in T2DM patients. The trial included a main study segment and a high glycemic substudy. The main study cohort comprised subjects inadequately managing their condition through diet and exercise alone, as well as those on antihyperglycemic agents (AHAs). Subjects who were not on AHAs underwent a placebo run-in period, whereas AHA users underwent an AHA washout followed by a placebo run-in. Eligible participants were men and women aged 18–80 with type 2 diabetes. In the main study, 587 patients were randomly assigned (1:1:1 ratio) to receive either canagliflozin at doses of 100 or 300 mg or placebo. Additionally, 91 subjects were enrolled in the high glycemic substudy because of elevated levels of HbA1c (10.0–12.0%) at screening or week −1. These individuals were subjected to a one-week, single-blind, placebo run-in period, followed by a 26-week, double-blind, active-treatment period. Randomization in the main study was stratified based on AHA use at screening and participation in the frequently-sampled mixed-meal tolerance test (FS-MMTT). Similarly, subjects in the high glycemic substudy were randomized to receive either canagliflozin 100 or 300 mg in a 1:1 ratio, with randomization stratified by AHA use at screening. After 26 weeks of treatment, both doses of canagliflozin demonstrated significant reductions in baseline HbA1c levels compared with the placebo, with reductions of 0.77% and 1.03% for the 100 mg and 300 mg doses, respectively. Notably, in the high glycemic cohort, canagliflozin elicited substantial reductions in baseline HbA1c levels, with reductions of 2.13% and 2.56% for the 100 mg and 300 mg doses, respectively. Furthermore, regardless of baseline use or history of treatment with AHAs, both cohorts exhibited similar reductions in HbA1c levels. Additionally, canagliflozin treatment resulted in notable reductions in body weight, systolic blood pressure (BP), and triglyceride levels while elevating high-density lipoprotein cholesterol levels compared with the placebo [14].

Canagliflozin was also studied in various placebo-controlled therapeutic combinations, including dual therapy with a sulfonylurea, adjunctive therapy with insulin, triple therapy alongside metformin and a sulfonylurea, triple therapy as an add-on to metformin and sitagliptin, and triple therapy combined with metformin and pioglitazone. Overall, canagliflozin consistently demonstrated clinically and statistically significant improvements in glycemic control and reductions in body weight compared with the placebo. Moreover, in triple therapy with metformin and pioglitazone and metformin and sitagliptin, a notable reduction in systolic BP associated with canagliflozin was observed [92,93,94,95,96].

In comparative trials in which an active control was used, canagliflozin was assessed against glimepiride in combination with metformin and against sitagliptin in conjunction with metformin and sulfonylurea [97,98]. Canagliflozin at a dosage of 100 mg, when used alongside metformin, resulted in comparable decreases in HbA1c levels from the baseline, while the 300 mg dosage demonstrated superior reductions in HbA1c compared with glimepiride, thus confirming its non-inferiority. Furthermore, canagliflozin not only demonstrated non-inferiority but also exhibited superiority over sitagliptin in lowering HbA1c levels in subsequent evaluations. Additionally, notable improvements in body weight and decreases in systolic blood pressure were observed when compared with both glimepiride and sitagliptin.

The Canagliflozin Cardiovascular Assessment Study (CANVAS) Program involved two parallel trials, CANVAS and CANVAS-R (renal), conducted from 2009 to 2015 across 667 centers in 30 countries. These trials enrolled approximately 10,000 participants with type 2 diabetes, targeting the evaluation of the impact of canagliflozin on cardiovascular and renal outcomes. Eligible participants had T2DM with HbA1c levels of 7.0–10.5%, were aged ≥ 30 years with symptomatic atherosclerotic vascular disease or were aged ≥ 50 years with at least two cardiovascular risk factors. In the CANVAS, participants were randomly assigned (1:1:1) to receive 300 mg canagliflozin, 100 mg canagliflozin, or matching placebo daily. In contrast, in the CANVAS-R, participants were randomly assigned (1:1) to receive canagliflozin or matching placebo at an initial dose of 100 mg daily, with the option to increase to 300 mg from week 13. The mean follow-up duration was 188 weeks. The primary outcomes from the CANVAS Program indicated the cardiovascular safety of canagliflozin showed a 14% reduction in major adverse cardiovascular events compared with the placebo. Exploratory analyses further revealed that among the patients treated with canagliflozin, 27% did not experience progression of albuminuria, while 70% showed a reduction in albuminuria levels. Additionally, there was a 40% decrease in a composite renal outcome, comprising sustained reductions in eGFR, end-stage kidney disease, or renal-related deaths. However, an elevated risk of amputation and fracture was noted with canagliflozin, irrespective of baseline kidney function. In summary, canagliflozin diminished the risk of sustained kidney function decline, reduced albuminuria, and moderated eGFR decline, suggesting its potential to alleviate kidney disease burden in individuals with type 2 diabetes [17].

Although canagliflozin’s efficacy in lowering blood glucose is reduced in patients with impaired renal function, interest in its renal-protective effects stemmed from early studies showing favorable outcomes in urinary albumin–creatinine ratio reduction and eGFR preservation. The CREDENCE (Canagliflozin and Renal Events in Diabetes with Established Nephropathy Clinical Evaluation) trial aimed to assess the advantages of canagliflozin use in preventing kidney failure and cardiovascular events while ensuring safety in high-risk type 2 diabetes patients with kidney disease progression. This double-blind, placebo-controlled trial involved 4401 participants with T2DM and CKD, randomly assigned to daily receive either oral canagliflozin 100 mg or placebo, with a median follow-up duration of 2.62 years. Eligible subjects were ≥30 years of age with T2DM, eGFR of 30–<90 mL/min/1.73 m^2^, and were receiving treatment with a stable maximum labeled or tolerated dose of angiotensin-converting enzyme inhibitor or angiotensin receptor blocker for ≥four weeks before randomization. By design, approximately 60% of participants were to have a screening eGFR of 30 to <60 mL/min/1.73 m^2^. Following the initial screening, participants were either directed to engage in a two-week single-blind placebo run-in or underwent further screening if deemed necessary for different reasons, such as having maintained a stable dose of renin-angiotensin blockade therapy for at least four weeks. Those who did not immediately enter the two-week run-in period underwent a repeated measurement of eGFR at the start of this phase. The trial demonstrated consistent benefits of canagliflozin across different eGFR subgroups, with lower eGFR subgroups experiencing more significant renal benefits. Canagliflozin did not increase the risk of serious adverse events, amputations, or fractures across eGFR subgroups. Overall, canagliflozin effectively reduced the risk of renal and cardiovascular events, with the greatest benefits observed in subgroups with lower eGFR [99].

Overall, canagliflozin demonstrated favorable tolerability, with predominant adverse events including genital and urinary tract infections, constipation, thirst, nausea, and polyuria. Notably, among individuals diagnosed with type 2 diabetes who exhibited either established cardiovascular disease or a minimum of two cardiovascular risk factors, the administration of canagliflozin was linked to an elevated likelihood of lower limb amputation, as evidenced in the CANVAS Program. Conversely, findings from the CREDENCE trial revealed no visible variance in the risk of lower limb amputations associated with the use of canagliflozin at a dosage of 100 mg (in comparison with the placebo). Authors of the CREDENCE program state that the reasons for the increased risk of lower limb amputation observed in the CANVAS Program remain unclear, but the overall safety profile in the CREDENCE program is otherwise consistent with the known adverse effects associated with canagliflozin [17,88,99].

### 2.3. Empagliflozin

Empagliflozin is another SGLT2 inhibitor used to reduce hyperglycemia in patients with type 2 diabetes. It works in a similar way to dapagliflozin and does not inhibit other glucose transporters important for glucose transport into peripheral tissues; it is 5000 times more selective for SGLT2 versus SGLT1. The drug was approved by both the FDA and EMA in 2014 and is being sold in combination with linagliptin (empagliflozin + linagliptin) and metformin (empagliflozin + metformin hydrochloride) [100,101,102,103]. 

A study conducted by Roden et al. [15] aimed to evaluate the efficacy and safety of empagliflozin in comparison with placebo and sitagliptin. A total of 899 patients were allocated to a 24-week treatment (1:1:1:1 for placebo, 10 mg empagliflozin, 25 mg of empagliflozin, 100 mg sitagliptin), and 87 patients were allocated to the open-label trial. Both doses of empagliflozin led to significant improvements in HbA1c, body weight, and systolic BP compared with the placebo or sitagliptin. The reductions in HbA1c were notably greater in patients with higher baseline HbA1c levels. Empagliflozin was well tolerated and had a good safety profile. It was not associated with an increased risk of hypoglycemia, which is a common concern with diabetes medications. However, urinary tract and genital infections have been reported more frequently with this drug, although most were mild and did not lead to discontinuation. Another study conducted by Häring et al. [104] also, through a 24-week period, checked the efficacy and tolerability of empagliflozin as an add-on to metformin therapy in patients with type 2 diabetes. Patients who received metformin (1500 mg/day) were randomized and treated with 10 mg/day empagliflozin, 25 mg/day empagliflozin, or placebo. The subjects treated with empagliflozin showed significant reductions in HbA1c levels compared with those treated with a placebo. Again, the reductions were greater in patients with higher baseline HbA1c levels. Additionally, empagliflozin treatment resulted in significant reductions in body weight and systolic blood pressure compared with the placebo. These two trials demonstrate that empagliflozin therapy effectively improves glycemic control, reduces body weight, and lowers systolic BP in patients with type 2 diabetes. 

Empagliflozin, in combination with linagliptin, has also demonstrated significant efficacy in managing T2DM, as evidenced by the findings of three clinical trials. In a phase 3 clinical trial [105], the combination of empagliflozin and linagliptin was compared with empagliflozin or linagliptin monotherapy in adults with insufficiently controlled diabetes. Following a 24-week treatment period, the results showed substantial improvements in HbA1c levels compared with monotherapy with either empagliflozin or linagliptin. Additionally, combination therapy resulted in a significant reduction in body weight. In another trial [106], participants receiving combination therapy experienced a greater reduction in HbA1c levels compared with the placebo or linagliptin monotherapy. Despite the higher rates of adverse events, the combination therapy demonstrated favorable efficacy outcomes without severe hypoglycemia cases reported. The last trial [107] further supports the efficacy of empagliflozin in combination with linagliptin. Participants treated with this combination also showed greater reductions in HbA1c levels than those receiving monotherapy. Moreover, the efficacy was sustained over a 52-week period, with no significant increase in adverse events compared with individual therapies.

Empagliflozin, in combination with metformin, has also demonstrated significant efficacy in the management of T2DM. In a trial managed by Ridderstråle et al. [108], which extended over a period of up to 104 weeks, empagliflozin was compared with glimepiride in patients with diabetes. The study revealed that empagliflozin, when used in combination with metformin, led to significant reductions in HbA1c levels compared with glimepiride, indicating better glycemic control over an extended treatment duration. Additionally, empagliflozin showed sustained reductions in body weight and blood pressure, along with preserved renal function. Notably, adverse event rates were comparable between the empagliflozin and glimepiride groups, with empagliflozin demonstrating a lower incidence of hypoglycemic adverse events. In another trial conducted by Hadjadj et al. [109], results at week 24 showed that empagliflozin/metformin combination therapy resulted in greater reductions in HbA1c levels than empagliflozin or metformin alone. Furthermore, empagliflozin plus metformin treatment led to significant reductions in body weight compared with metformin alone. This combination therapy offers a promising treatment option for individuals requiring additional glycemic management beyond metformin monotherapy.

Rosenstock et al. managed two trials assessing the safety and efficacy of empagliflozin treatment in patients inadequately controlled on their current diabetes treatment (patients inadequately controlled on basal insulin [110] and on multiple daily injections of insulin [111]). In the first trial at week 18, both studied doses of empagliflozin (10 and 25 mg) led to significant reductions in HbA1c levels compared with the placebo. This improvement was sustained through week 78, with empagliflozin also significantly reducing the insulin dose and body weight compared with the placebo. Additionally, empagliflozin 10 mg significantly reduced systolic BP compared with the placebo. Confirmed hypoglycemic events were similar across all treatment groups; however, higher rates of urinary tract and genital infections were reported in patients treated with empagliflozin. In the second trial, both doses of empagliflozin (also 10 and 25 mg) led to significant reductions in HbA1c levels compared with the placebo at week 18, with further reductions observed at week 52. More patients achieved HbA1c levels below 7.0% with empagliflozin compared with the placebo. Empagliflozin also reduced insulin doses and body weight compared with the placebo at week 52.

In addition to the aforementioned studies, three additional trials investigating the efficacy and safety of empagliflozin in patients with various stages of renal impairment were conducted [18,19,20]. Most of the patients also suffered from type 2 diabetes; only one study conducted by The EMPA-KIDNEY Collaborative Group [19] allowed non-diabetic patients, and they made up about 50% of the whole group. The results showed that empagliflozin effectively reduced HbA1c levels compared with the placebo in patients with kidney issues in all three trials, demonstrating its efficacy across different levels of CKD severity. Additionally, these trials showed that empagliflozin provided advantages beyond glycemic control, including reducing the risk of cardiovascular events, slowing CKD progression, and stabilizing eGFR. These benefits were observed across various KDIGO (Kidney Disease: Improving Global Outcomes) risk categories and were maintained over the treatment periods. Although empagliflozin was generally well tolerated, there were some notable adverse events [18,20]. These included genital and urinary tract infections. The incidence of adverse events varied depending on the stage of chronic kidney disease, with higher rates observed in patients with stage 4 CKD. Despite the occurrence of adverse events, the overall safety profile of empagliflozin was consistent across the trials. Serious adverse events were infrequent, and there were no apparent increases in the incidence of adverse events compared with the placebo, except for genital infections. In conclusion, the trials collectively demonstrate that empagliflozin offers significant benefits in glycemic control, cardiovascular and renal protection, and an overall safety profile in patients with kidney problems across different stages of CKD, with or without diabetes.

The efficacy and safety of empagliflozin in patients with chronic heart failure with a left ventricular ejection fraction of ≤40%, regardless of the presence or absence of diabetes, were also examined in a trial supervised by Packer et al. [22]. This trial aimed to include adults aged 18 years or older with chronic heart failure who were at increased risk of serious heart failure events. The patients received standard heart failure treatment. The primary outcome was a composite of cardiovascular death or hospitalization due to heart failure. A total of 3730 patients were randomly assigned to receive either empagliflozin or a placebo. Empagliflozin showed a significant reduction in the primary outcome compared with the placebo. This effect remained consistent across various patient subgroups. Empagliflozin also reduced heart failure hospitalizations and slowed the decline in kidney function. Additionally, composite renal outcomes occurred less frequently with empagliflozin. There was no significant difference in the overall mortality between the empagliflozin and placebo groups. However, uncomplicated genital tract infections were more common with empagliflozin treatment. 

### 2.4. Ertugliflozin

Ertugliflozin is a potent, selective, and reversible inhibitor of SGLT2 that functions in a manner similar to dapagliflozin. It was registered by EMA and FDA [112,113] in 2017–2018. Furthermore, it is accessible in combination with either metformin or sitagliptin [114,115]. Dose–response modeling indicates a nearly maximal urinary glucose excretion response at dosages of 5 and 15 mg. Peak plasma concentrations of ertugliflozin are observed one-hour post-dosage under fasted conditions. Steady-state is achieved within four to six days of once-daily administration, and the pharmacokinetic characteristics of the drug remain consistent over time. The absolute oral bioavailability of ertugliflozin following the administration of a 15 mg dose is approximately 100% [112].

The clinical efficacy and safety profile of ertugliflozin were evaluated in the VERTIS trial (eValuation of ERTugliflozin effIcacy and Safety), which consisted of seven individual studies. VERTIS MONO, a 52-week, double-blind, multicenter, randomized, parallel-group study comprising a 26-week placebo-controlled treatment period (phase A), succeeded by a 26-week active-controlled treatment period (phase B), involved 461 adult participants experiencing inadequate glycemic control (HbA1c 7.0–10.5% inclusive) despite diet and exercise regimens. The primary outcome measure was the alteration in HbA1c levels from baseline to week 26. These individuals, who were not undergoing any concurrent antihyperglycemic treatment, were randomly assigned to receive either ertugliflozin 5 mg, ertugliflozin 15 mg, or placebo once daily. Treatment with both 5 and 15 mg doses of ertugliflozin exhibited efficacious glycemic control and led to reductions in body weight [16,116]. The advantages of ertugliflozin remained generally consistent throughout week 52 of the VERTIS MONO trial, following an additional 26-week phase under active control [117]. 

The investigation of ertugliflozin in conjunction with metformin was conducted within the VERTIS MET trial. This double-blind, multicenter study spanned 26 weeks and involved a total of 621 participants exhibiting inadequate glycemic control while on metformin monotherapy. These participants were randomly assigned in a 1:1:1 ratio to receive either placebo or ertugliflozin at doses of 5 or 15 mg/day. The primary endpoint focused on the change in HbA1c levels from baseline to week 26. The addition of ertugliflozin to metformin treatment among patients with insufficiently controlled T2DM demonstrated enhancements in glycemic regulation and reductions in body weight and BP, although accompanied by an elevated occurrence of genital mycotic infections [118]. 

The VERTIS SU trial investigated the comparative effectiveness of ertugliflozin versus glimepiride as a supplementary therapy alongside metformin in patients diagnosed with T2DM. This double-blind, multicenter study spanned 104 weeks and employed an active comparator-controlled design, randomly allocating (1:1:1) a total of 1326 inadequately controlled T2DM patients on metformin monotherapy to receive once-daily either ertugliflozin at doses of 5 mg or 15 mg, or glimepiride, while continuing background metformin therapy. Glimepiride initiation started at 1 mg/day and was gradually titrated up to a maximum dosage of either 6 or 8 mg/day (depending on the maximum approved dose in each country) or a dosage determined by individual tolerability, with a mean daily dose of 3 mg. After 52 weeks of treatment, ertugliflozin at a dosage of 15 mg was non-inferior to glimepiride in reducing HbA1c levels when used in conjunction with metformin in T2DM patients. Both doses of ertugliflozin exhibited clinically significant improvements in glycemic control, alongside reductions in body weight and blood pressure [119].

A factorial study with ertugliflozin and sitagliptin as add-on combination therapy with metformin (VERTIS FACTORIAL) aimed to assess the efficacy and safety of ertugliflozin 5 mg or 15 mg in combination with sitagliptin 100 mg, compared with the individual components. A total of 1233 individuals diagnosed with T2DM who experienced inadequate glycemic regulation while undergoing metformin monotherapy (≥1500 mg/day) were subjected to randomization into one of five active treatment groups: ertugliflozin at dosages of 5 mg or 15 mg, sitagliptin at 100 mg, or sitagliptin at 100 mg combined with either 5 mg or 15 mg of ertugliflozin, administered once daily, alongside continuation of background metformin therapy. The primary efficacy endpoint was centered on assessing alterations in HbA1c levels from baseline to week 26. In individuals with poorly managed T2DM while on metformin treatment, the co-administration of ertugliflozin and sitagliptin resulted in superior glycemic control over the course of 52 weeks compared with the individual agents. Furthermore, this combined therapy led to reductions in body weight and systolic BP over a 52-week duration in comparison with treatment with sitagliptin alone [120]. 

Otherwise, the VERTIS SITA trial evaluated the efficacy of combined therapy comprising either ertugliflozin at doses of 5 mg or 15 mg once daily along with sitagliptin 100 mg/day to achieve effective glycemic control among patients diagnosed with T2DM, whose glycemic levels remained inadequately managed through diet and exercise. This phase 3, randomized, double-blind, multicenter, placebo-controlled study encompassed a duration of 26 weeks and involved 291 T2DM patients with HbA1c levels ranging from 8.0% to 10.5% at baseline despite dietary and exercise regimens. Participants were randomized in a 1:1:1 ratio to receive either ertugliflozin 5 mg and sitagliptin 100 mg, ertugliflozin 15 mg and sitagliptin 100 mg, or placebo once daily. The primary endpoint assessed was the alteration in HbA1c levels from baseline to week 26. Noteworthy reductions in body weight and systolic blood pressure were also evident in both groups receiving combined ertugliflozin/sitagliptin therapy compared with the placebo group [121]. 

Furthermore, in the VERTIS SITA2 trial, the addition of ertugliflozin to existing metformin and sitagliptin doses demonstrated favorable tolerability and contributed to clinically meaningful and sustained improvements in glycemic control, alongside reductions in body weight and systolic BP, over a period of 52 weeks. This double-blind, randomized study involved 464 participants with HbA1c levels of 7.0–10.5%, who were receiving metformin at a dosage of ≥1500 mg/day and sitagliptin at 100 mg/day. The participants were randomly assigned to receive ertugliflozin at doses of 5 mg once daily, 15 mg once daily, or the placebo. The primary efficacy endpoint was the change in HbA1c levels from baseline at week 26, with treatment continuation until week 52. Notably, ertugliflozin treatment was associated with a higher incidence of genital mycotic infections in both male and female participants than in the placebo group [122].

VERTIS CV (Evaluation of Ertugliflozin Efficacy and Safety Cardiovascular Outcomes Trial), a randomized, double-blind, multicenter study conducted in T2DM patients with concurrent cardiovascular disease, notably included a substantial proportion of participants with a history of heart failure and documented pre-trial ejection fraction. A total of 8246 individuals diagnosed with T2DM and atherosclerotic cardiovascular disease were subjected to random assignment into one of three groups receiving once-daily doses of either ertugliflozin at 5 mg, 15 mg, or placebo. The primary endpoint of the trial focused on assessing the duration until the occurrence of the initial major adverse cardiovascular event. Ertugliflozin treatment reduced both the occurrence of the first hospitalization for heart failure events and the overall frequency of such events. This reduction in risk was consistently favorable in individuals with and without a history of heart failure and among those with heart failure history, irrespective of whether their ejection fraction was ≤45% or >45%. While the impact of ertugliflozin on the risk of initial hospitalization for heart failure remained consistent across most baseline subgroups, heightened efficacy of ertugliflozin was particularly notable in three specific populations: those with an estimated glomerular filtration rate < 60 mL/min/1.73 m^2^, individuals presenting with albuminuria, and those treated with diuretics. The findings from this investigation further substantiate the advantage of SGLT2 in both primary and secondary prevention strategies aimed at addressing heart failure events in patients with T2DM [23]. 

The safety and efficacy of ertugliflozin were also evaluated over 52 weeks in patients with CKD in the VERTIS RENAL trial. This double-blind, randomized study involved 468 subjects with HbA1c levels ranging from 7.0% to 10.5% and stage 3 CKD who were receiving standard diabetes therapy, such as metformin, insulin, and/or sulfonylureas. Participants were randomly assigned to receive once-daily doses of either ertugliflozin at 5 mg, 15 mg, or placebo. Patients treated with metformin underwent a wash-off period of at least 10 weeks before randomization. The primary endpoint was the change in HbA1c levels from baseline to week 26. Approximately 17% of patients were found to have engaged in prohibited metformin use, which affected the assessment of the primary endpoint. Despite the influence of covered metformin use on the primary analysis, reductions in both blood glucose levels and body weight were observed with ertugliflozin among patients diagnosed with T2DM and stage 3 CKD while demonstrating an acceptable safety profile [21]. 

Common adverse reactions to ertugliflozin include genital and urinary tract infections, increased urination, and thirst [112]. 

### 2.5. Sotagliflozin

Sotagliflozin was approved by the FDA in May 2023. This drug is intended to reduce the risk of cardiovascular death, hospitalization for heart failure, and urgent heart failure visits in adults with heart failure, type 2 diabetes mellitus, chronic kidney disease, and other cardiovascular risk factors [123]. Despite the fact that the drug was approved by the FDA in Europe, the EMA has withdrawn its marketing authorization for sotagliflozin [124].

Table 2 summarizes the clinical information on the safety and clinical application of flozins, with a focus on adverse events.

## 3. Myrcludex B

Sodium taurocholate co-transporting polypeptide (NTCP), encoded by the solute carrier family 10 member 1 (SLC10A1) gene, is a transmembrane sodium-dependent uptake transporter located in the basolateral membrane of human hepatocytes and plays an important role in bile salts hepatic uptake [24]. It consists of nine transmembrane segments (TMs): three of them (TM1, TM5, and TM6) model ‘panel’ domain on the N-terminal edge, while TM2, TM3, TM4, TM7, TM8, and TM9 form a six-TM ‘core’ domain [125]. Studies have revealed that TMs form a polarized tunnel connecting the extracellular environment with the hepatocyte cytoplasm, where the external side consists of hydrophilic residues, whereas aliphatic and aromatic side chains on the internal part of the tunnel create a hydrophobic surface [126]. NTCP, apart from transporting bile salts, is a key element in the cellular entry of the hepatitis B virus (HBV) and hepatitis delta virus (HDV) particles into hepatocytes. The viral Pre-S1 domain of large envelope surface protein has been identified as the main structure of HBV in attaching and entering human cells [25]. HDV is a viral entity that has been identified as defective, as it does not possess its own envelope proteins. Instead, it co-opts the envelope proteins of HBV, particularly the Pre-S1 domain, for its assembly and propagation [26]. A correlation between the extracellular region of TM5 of NTCP, specifically residues 157–165, and the attachment of HBV was established. This region of NTCP is situated near the tunnel entry and is thought to play a key role in the process of viral attachment [24], with a single amino acid at position 158 being crucial [25]. Another critical NTCP binding region is formed by residues 84–87, located on the TM2–TM3 loop in the core domain [125]. The Pre-S1 binding is also a myristoylation-dependent process, and research has shown that myr-Pre-S1 has approximately 1000-fold higher inhibitory activity than unmodified Pre-S1 [24]. Furthermore, Pre-S1 binding interferes with substrate transport, which indicates that the Pre-S1 attached to NTCP may overlap with the substrate-binding site. It has been hypothesized that the myristoyl moiety is translocated from the virus to the hepatocyte membrane, but the exact spatial coordinates of the moiety are unknown [125].

It is well-established that HDV can only be transmitted in the presence of HBV, and it is estimated that approximately 15% of HBV-positive patients have coexisting HDV infection. The coinfection is often more severe than the HBV infection itself and can result in chronic HDV. Patients with chronic HDV are two times more likely to develop and die from hepatocellular carcinoma than those with mono-infection [26]. 

**Figure 2:**
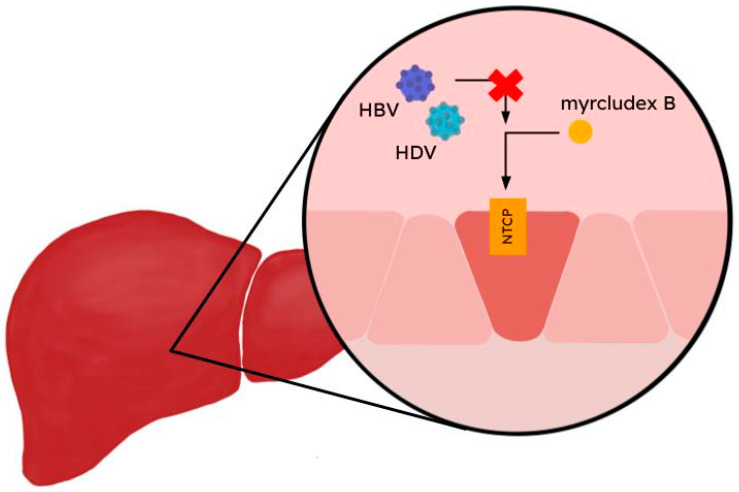
Mechanism of action of myrcludex B in hepatocytes. Myrcludex B competes with hepatitis B virus (HBV)/hepatitis D virus (HDV) for sodium taurocholate co-transporting polypeptide (NTCP) and consequently inhibits the entry of the virus into cells.

As NTCP plays an important role in the virus particle entry, it can be considered for the development of therapeutic agents. The blocking process of viral entry results in the inhibition of the development of the disease because of the inability to replicate viral RNA outside the hepatocytes and triggers the development of a linear, synthetic myristoylated lipopeptide consisting of 47 amino acids in the Pre-S1 region called myrcludex B (Figure 2) [25]. Myrcludex B, registered in Europe to treat HDV-CH, was designated by the European Commission as an ‘orphan medicine’ on 19 June 2015 (EU/3/15/1500) [127]. Its antiviral properties may be linked to two mechanisms of action: one involves competition with HBV/HDV for the NTCP binding site, and the other involves inhibition of RNA transcription inside cells [128]. In a phase 2b trial conducted by Wedemeyer et al. [129], the safety and efficacy of myrcludex B combined with tenofovir (registered HBV/HDV infection drug) was evaluated. A total of 120 participants with chronic HBV/HDV coinfection were randomized into four groups—tenofovir (245 mg/day for 12 weeks) with 2 mg (A), 5 mg (B), or 10 mg (C), myrcludex B (for 24 weeks, followed by only tenofovir again for 24 weeks) and tenofovir alone (D). At the end of the treatment, HDV RNA reduction by 2log10 from the baseline was reached by 46.4%, 46.8%, 76.6%, and 3.3% of patients in Groups A, B, C, and D, and the median HDV RNA serum levels declined by −1.75 log, −1.60 log, −2.70 log and −0.18 log in each group respectively [128,129,130]. Pegylated interferon-alpha (PEG-IFN-α) is another drug used to treat chronic HBV/HDV coinfections. A randomized phase 2 trial by Wedemeyer et al. [131] tested the efficacy of combination treatment and monotherapy in a 48-week period. Patients with chronic infection were divided into four groups: 2 mg myrcludex B (A), 180 µg PEG-IFN-α, (B), 2 mg myrcludex B + PEG-IFN-α (C), and 5 mg myrcludex B + PEG-IFN-α (D). The log10 changes in median HDV RNA in serum at week 48 were as follows: −2.84 log10 (A), −1.14 log10 (B), −3.62 log10 (C), and −4.48 log10 (D). Only two patients (13%) in both A and B groups became HDV RNA-negative at the end of the treatment, while 15 patients (50%) became so with the combination treatment (Groups C and D) [128,130,131].

The ongoing randomized phase 3 study run by Wedemeyer et al. [127] aims to investigate the long-term effects of myrcludex B therapy. A total of 150 patients were randomized into three groups: 2 mg myrcludex B for 144 weeks (A), 10 mg myrcludex B for 144 weeks (B), and no treatment for 48 weeks, followed by 10 mg myrcludex B for 96 weeks (C) are recruited. As this trial is still in progress, only the results after a 48-week period were published. HDV RNA levels in serum at week 48 were undetectable in 12% (A) and 20% (B) of patients. ALT (alanine aminotransferase) levels were also measured, and its normalized levels were observed in 51% (A), 56% (B), and 12% (C) of patients.

In these studies, myrcludex B was well tolerated; common side effects included headache, pruritus, fatigue, eosinophilia, injection-site reactions, upper abdominal pain, arthralgia, and asthenia [127]. Slight, asymptomatic increases in bile acids were also observed, but they returned to baseline levels after treatment discontinuation [127,129,131]. These results suggest an additive or synergistic effect of combination treatment with myrcludex B and either tenofovir or PEG-IFN-α. Myrcludex B also showed dose-dependent antiviral efficacy against HDV. Although the results are promising, further validation with longer treatment durations or maintenance therapy studies is required.

## 4. Odevixibat and Maralixibat

Apical sodium-dependent bile salt transporter (ASBT) is a product of SLC10A2 gene expression, with 35% identity and 63% amino acid sequence similarity with NTCP [30]. Similarly, the main function of NTCP is the sodium-dependent uptake of bile salts. While NTCP is expressed in hepatocytes, ASBT is mainly expressed on the apical membrane of enterocytes in the terminal ileum [30], but it can also be found in the kidneys and cholangiocytes [27]. ASBT is responsible for up to 95% of bile salts uptake from the ileum [28] by selectively transporting mainly conjugated bile acids but also showing affinity towards unconjugated bile acids [27,29].

ASBT consists of 348 amino acids with a molecular weight of approximately 43 kDa [30]. Studies have shown that the ASBT structure is similar to that of NTCP; it has seven transmembrane segments with an amino terminus on the extracellular site and a carboxyl terminus at the cytoplasmic site [132]. Studies have also identified four distinct substrate-binding sites, but further research is needed to acquire precise information [30].

Impaired bile formation and flow (i.e., cholestasis) can lead to pruritus, liver inflammation, or injuries and can be fatal. Alagille syndrome (ALGS) and Progressive Familial Intrahepatic Cholestasis (PFIC) are examples of inherited cholestatic diseases. ALGS is an autosomal dominant disease caused by mutations or deletions in JAGGED1 (more than 90% of cases) or NOTCH2 genes [28,31]. PFIC is a group of diseases caused by autosomal recessive mutations in bile acid membrane transporter genes [28,32]. Recent studies have reported six types of PFIC based on the observed mutant genes: PFIC1 (ATP8B1), PFIC2 (ABCB11), PFIC3 (ABCB4), PFIC4 (TJP2), PFIC5 (NR1H4), and PFIC6 (associated with MYO5B defects) [32].

Both ALGS and PFIC patients can suffer from a reduced quality of life due to symptoms, hospital visits, and the requirement for extensive long-term care, which can significantly affect their physical and mental health [28]. Many patients need liver transplantation; the estimated liver transplant-free survival in ALGS patients was 24% at the age of 18.5 [133], while observed native liver survival at 18 years of age for PFIC1 and PFIC2 patients was 51% and 32%, respectively [134]. Currently, no medication has been approved specifically for the treatment of either ALGS or PFIC. Instead, medical interventions generally focus on reducing symptoms [28]. One of the most frequent and burdensome symptoms is pruritus, which can be associated with scratching-induced scarring, sleep disturbances, emotional disturbances, suicidal ideation, and overall disruption of quality of life [28,135]. Pruritus shows a circadian rhythm where itching becomes more severe in the evening; however, other factors such as stress, heat, the progesterone phase of the menstrual cycle, and contact with wool can also contribute to increased itching [135,136]. There are many components, such as endogenous opioids, steroids, and lysophosphatidic acid with autotaxin enzyme, which play a role in the occurrence and severity of cholestatic pruritus, and its pathogenesis is yet to be elucidated [135]. Elevated levels of bile salts in the serum are one of the factors often present in cholestatic patients. Although there is no correlation between the severity of the itch and the concentration of bile salts in serum, pruritus can also be observed in patients with normal levels of bile salts. The FDA and EMA approved two drugs that can decrease bile salt levels in serum, thus reducing the severity of pruritus in pediatric patients [28,135,136].

**Figure 3:**
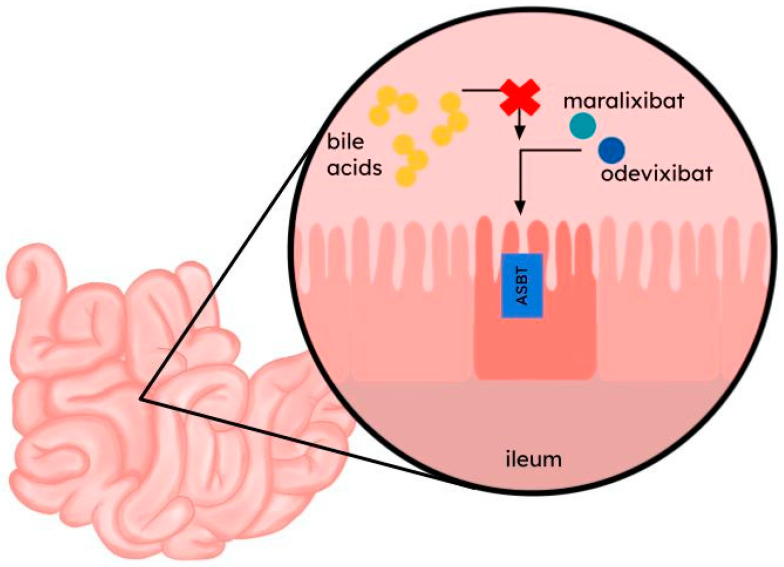
Mechanism of action of maralixibat and odevixibat. Maralixibat and odevixibat bind selectively with apical sodium-dependent bile salt transporter (ASBT), inhibiting the reuptake of bile salts in the distal ileum, thus reducing bile salts resumption to the liver.

Odevixibat and maralixibat are selective, reversible, small-molecule inhibitors of the apical sodium-dependent bile salt transporter (Figure 3) [137,138]. ASBT inhibition results in decreased reuptake of bile salts in the distal ileum, which reduces bile salts return to the liver, thus reducing their concentration in serum [28]. There is no substantial evidence indicating that they inhibit NTCP.

Two major phase 3 trials were conducted to evaluate the safety and efficacy of odevixibat in PFIC patients. In the PEDFIC I led by Thompson et al. [139], 62 patients aged 0.5—18 years with PFIC1 or PFIC2 were stratified into three groups: odevixibat 40 μg/kg/day (A), odevixibat 120 μg/kg/day (B), and placebo (C). In a sample of 14 patients (33.3% of all participants) who were monitored for 24 weeks, it was observed that 10 patients in Group A, four in Group B, and zero in Group C demonstrated a reduction in serum bile salts of at least 70% from their initial levels. Additionally, a decrease in the severity of pruritus was noted, with 58.3% of patients in Group A, 47.7% in Group B, and 28.7% in Group C reporting less severe itching and requiring fewer days of medical intervention for relief. These patients also experienced improved sleep quality, with less difficulty falling asleep and sleeping more soundly with the assistance of their guardians.

The PEDFIC II study conducted by Thompson et al. [140] is an ongoing, open-label 72-week study. Patients were divided into two cohorts: one with patients from PEDFIC I and two with newly recruited patients of any age and any PFIC type (*n* = 16). Cohort 1 was further divided into two groups: 1A—patients treated with odevixibat in PEDFIC I (*n* = 34) and 1B—patients treated with the placebo in PEDFIC I (*n* = 19). During the PEDFIC II study, all patients were treated with odevixibat 120 μg/kg/day. A total of 45 patients were evaluated after at least 48 weeks of odevixibat treatment, including 13 patients with PFIC1, 30 with PFIC2, 1 with PFIC3, and 1 with PFIC6. A lasting effect on the reduction of serum bile acids, as well as a reduction in ALT, AST (aspartate aminotransferase) activities, and total bilirubin levels, was observed. Positive pruritus assessments in the first 24-week period of PEDFIC II were reported by 33%, 56%, and 62% of patients in Cohorts 1A, 1B, and 2, respectively. Long-term treatment with odevixibat was well tolerated and was associated with sustained reductions in serum bile salt levels and pruritus. The majority of the adverse effects were mild or moderate, self-limiting, and considered by the investigator as not related to the study drug; no serious adverse effects related to odevixibat treatment were reported [139,140,141]. 

A placebo-controlled phase 2b study in children (aged one to 18 years) with Alagille syndrome named ICONIC, managed by Gonzales et al. [137], was conducted to assess the efficacy and safety of maralixibat treatment. A total of 31 children with ALGS (JAGGED1 mutation) cholestasis and pruritus were first treated with maralixibat 380 μg/kg/day for 18 weeks. Subsequently, 29 patients who positively ended the first phase were randomized into two groups: those who continued maralixibat treatment (Group A, *n* = 13) and those who received a placebo (Group B, *n* = 16) for four weeks (withdrawal phase). Ultimately, all patients resumed maralixibat treatment at a dose of 380 μg/kg/day for up to 48 weeks. In the first 18 weeks, serum bile acid levels and pruritus severity decreased. In the withdrawal phase, participants who switched to placebo had significant increases in serum bile acid levels and severity of pruritus, while children who continued maralixibat maintained the treatment effect. When patients in the placebo group restarted maralixibat treatment after completing the withdrawal phase, serum bile acid levels decreased to levels previously observed during the first maralixibat treatment.

These data suggest that ASBT inhibitors such as maralixibat and odevixibat, which interrupt enterohepatic circulation, may be safe and efficacious treatment options for patients with ALGS and PFIC.

## 5. Probenecid and Benzbromarone

Antibiotics such as penicillins and ciprofloxacin are well-known substrates of organic anion transporters 1 and 3 (OAT1 and OAT3). These transporters play an important role in the renal excretion of penicillins, contributing to lowering their blood concentrations [33]. OAT1 and OAT3, along with urate transporter 1 (URAT1), are organic anion transporters of the SLC22 family [34]. Human OAT1 and OAT3 are mainly expressed in the basolateral membrane of the proximal tubule cells in the kidney [36]; however, they are also found in the brain, choroid plexus, and spinal cord [34,37]. Human URAT1 is also expressed in proximal tubule cells, but it is located in the apical membrane [40]. Although URAT1 is mainly expressed in the kidney, it can also be found in the heart, liver, prostate, skeletal muscles, small intestine, colon, adrenal gland, and pituitary gland [41]. OAT1 and OAT3 play an important role in the renal excretion of urate and other metabolites, such as endogenous anions and many anionic drugs, including β-lactam antibiotics, ciprofloxacin or some non-steroidal anti-inflammatory drugs. These transporters mediate the translocation of urate across the basolateral membrane [35], which can stimulate urate/anion antiport via URAT1 at the luminal membrane, resulting in increased urate reabsorption [34]. 

OAT1/3 inhibitors disrupt the tubular secretion of their substrates. In 1950, Beyer et al. [142] introduced p-(di-n-propylsulfamyl)-benzoic acid, also known by its generic name as probenecid. After oral or intravenous administration, this lipid-soluble molecule is absorbed from the intestinal tract and binds to 85–95% of plasma proteins, especially albumin. It can also diffuse freely across the blood–brain barrier and be actively transported out of the brain [143,144]. According to studies, administration of 2 g per day of probenecid orally has been found to significantly increase levels of penicillin in the bloodstream, with some research indicating an increase of up to five times the original concentration [145]. Historically, probenecid was co-administered with penicillin in World War II in order to decrease its overall renal secretion and reduce the administered dose. However, since the 1950s, antibiotic production has become easier, cheaper, and safer, and the clinical relevance of probenecid in this field has decreased [146]. Nevertheless, summaries of drug characteristics still include information on the above drug-probenecid interaction since probenecid-boosted β-lactam therapy is associated with improved outcomes, especially in gonococcal disease [147]. 

The interaction of probenecid with OAT1 is also used in clinical routine to reduce the nephrotoxic potential of cidofovir, an antiviral agent. OAT1-mediated uptake of cidofovir results in selective accumulation and toxicity in renal proximal tubular cells. Probenecid, a known inhibitor of OAT1, is used clinically in conjunction with cidofovir in order to reduce its accumulation in renal proximal tubular cells and prevent its toxicity [148]. This therapeutic strategy is registered for the treatment of cytomegalovirus retinitis in patients with acquired immunodeficiency syndrome and without renal dysfunction. 

**Figure 4:**
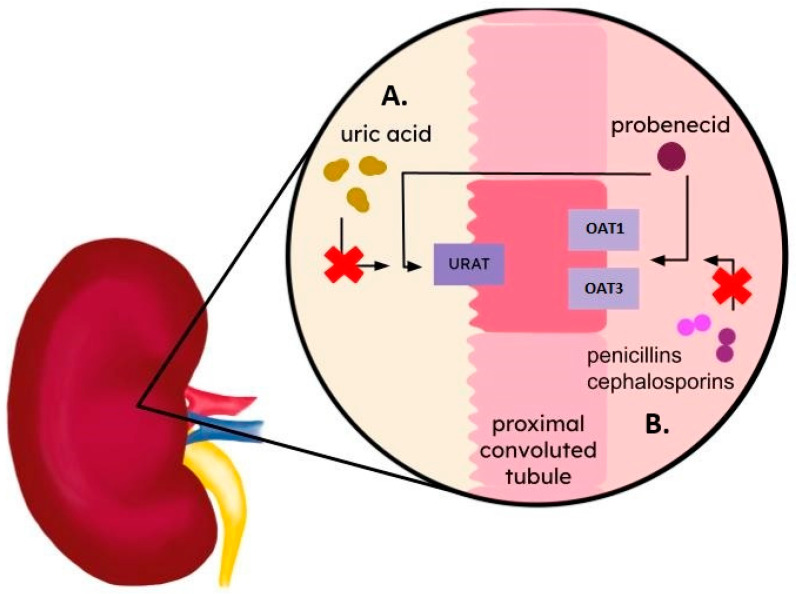
Different mechanisms of action of probenecid in the proximal renal tubule. A. Uric acid reuptake inhibition by binding with urate transporter 1 (URAT1), which enhances the excretion of urate in the kidney; B. Inhibition of penicillin and cephalosporin excretion by binding with organic anion transporters 1 and 3 (OAT1/3) and contributing to increased levels of these drugs in the bloodstream.

Probenecid has also been used since the 1950s to treat gout flares [38,39]. Gout is characterized by joint and surrounding tissue inflammation due to monosodium urate crystal deposition, leading to swelling and pain. One of the most frequent types of inflammation occurs in the first metatarsophalangeal joint. However, other joints, such as the ankle or midfoot, can also become inflamed [149]. One possible approach to treat gout is to reduce serum urate levels, which can help dissolve the monosodium urate crystals. Probenecid enhances the excretion of urate in the kidney by inhibiting OAT1/3 and URAT1 (Figure 4) [150]. It can also be used in patients with chronic kidney disease with mild to moderate renal impairment, particularly if no other treatment option is available [151]. Despite its effectiveness, probenecid is no longer as popular as it was in 20th century due to colchicine and steroids taking up the role of primary drugs in the acute phase of gout and the development of other drugs, such as allopurinol or febuxostat [146]. However, in 2018, Seoyoung et al. [152] reported that probenecid was associated with a 20% lower risk of hospitalization for myocardial infarction or stroke than allopurinol but only a 9% lower risk of hospitalization for heart failure, concluding from a large cohort study on 38,888 elderly patients with gout, who had an increased risk of cardiovascular diseases such as myocardial infarction, stroke or heart failure. 

Probenecid was FDA-approved, is available also in combination with colchicine, and has been shown in multiple studies to have no significant side effects [45]. In Europe, it can be approved via national procedures; at present, it is available, for example, in Germany and Switzerland [46,47]. Another drug used in the treatment of gout is lesinurad, which functions as a selective uric acid reabsorption inhibitor by inhibiting the URAT1 transporter [42]. Although it is FDA-approved, it is currently not available for clinical use.

Benzbromarone is another potent uricosuric agent affecting the function of URAT1 and probably OAT1 (but not OAT3) in the proximal tubular cells [43]. Nowadays, it is used to treat gout in only several countries (Brazil, New Zealand, and some European countries). The drug was never approved in the United States and was widely withdrawn from the market by its manufacturer because of reported hepatotoxicity. Several studies and meta-analyses demonstrated the efficacy of the drug in reducing uric acid levels and preventing gout flares. Benzbromarone was shown to increase urate clearance by 500%, leading to an average 25–50% reduction in uric acid levels [43,44]. Benzbromarone was evidenced to possess a better efficacy in lowering the serum uric acid than probenecid [153]. The drug also exhibits similar or superior efficacy compared with allopurinol in reducing serum uric acid, and no difference in the success rates for gout treatment between the two drugs after dose scaling [154,155]. Overall, evidence suggests that benzbromarone and allopurinol are characterized by similar efficacies in reducing gout flares and tophi reduction, but benzbromarone has a faster rate of uric acid reduction [156]. Benzbromarone was also evidenced to affect the progression of chronic kidney disease. A comparison of benzbromarone (88.9 mg/day), allopurinol (100.0 mg/day), and febuxostat (40.0 mg/day) in a thirteen-year cohort of patients with chronic kidney disease revealed that benzbromarone was the most effective in reducing progression to dialysis. Furthermore, benzbromarone and febuxostat demonstrated reduced progression to end-stage kidney disease in comparison with allopurinol [157]. 

Other drugs targeting drug transport are less widely used in clinical routines but can offer added value in the treatment of less common disease states due to specific modes of action.

## 6. Conclusions

Understanding the mechanisms involved in the overall transport process and identifying substrates and inhibitors of drug transporters is crucial in developing drugs that target membrane transporters, which is a promising strategy for combating diseases. Implementation of agents targeting membrane transporters, especially SGLT2 inhibitors, resulted in changes in clinical guidelines for the treatment of common diseases, such as diabetes mellitus and heart failure. Drugs that target specific transporters may offer new types of targeted therapy. However, due to the wide tissue distribution of several transporters, some agents lack selective activity, and their clinical application was not reached, e.g., P-glycoprotein 1 (P-gp) inhibitors. Many other efflux and uptake transporters and carriers remain potential therapeutic targets, and efforts to define interacting agents are ongoing [158,159].

## Figures and Tables

**Table 1:** SLC transporters discussed in this review: nomenclature, substrates, localization, associated diseases/disorders and drugs.

Transporter Family	Transporter Protein	Predominant Substrates	Localization	Associated Diseases/Disorders	Corresponding Drugs
SLC5 (solute carrier 5)	SGLT2 (sodium–glucose transport protein 2)	Glucose [11]	Early proximal tubule of the kidney [12]	Type 2 diabetes mellitus (T2DM) [13,14,15,16] chronic kidney disease (CKD) [17,18,19,20,21], heart failure [22,23]	Dapagliflozin; canagliflozin; empagliflozin; ertugliflozin; sotagliflozin
SLC10 (solute carrier 10)	NTCP (sodium taurocholate co-transporting polypeptide)	Bile salts [24]	Liver, pancreas [24]	Hepatitis B virus (HBV) and hepatitis delta virus (HDV) coinfection [25,26]	Myrcludex B
	ASBT (apical sodium-dependent bile salt transporter)	Bile salts [27,28,29]	Terminal ileum, kidney, cholangiocytes [27,30]	Alagille syndrome (ALGS), Progressive Familial Intrahepatic Cholestasis (PFIC) with associated cholestasis and pruritus [28,31,32]	Odevixibat; maralixibat
SLC22 (solute carrier 22)	OAT1 (organic anion transporter 1)	Uric acid; endogenous anions, anionic drugs incl. β-lactam antibiotics, ciprofloxacin, and some non-steroidal anti-inflammatory drugs [33,34,35]	Proximal tubule of the kidney (basolateral membrane), brain, choroid plexus, and spinal cord [34,36,37]	Gout [38,39]	Probenecid; benzbromarone
	OAT3 (organic anion transporter 3)	Uric acid; endogenous anions, anionic drugs incl. β-lactam antibiotics, ciprofloxacin, and some non-steroidal anti-inflammatory drugs [33,34,35]	Proximal tubule of the kidney (basolateral membrane), brain, choroid plexus, and spinal cord [34,36,37]	Gout [38,39]	Probenecid
	URAT1 (urate transporter 1)	Uric acid [34]	Proximal tubule of the kidney (apical membrane), heart, liver, prostate, skeletal muscles, small intestine, colon, and adrenal gland. pituitary gland [40,41]	Gout [38,39,42,43,44,45,46,47]	Probenecid; lesinurad; benzbromarone

**Table 2:** Overview of clinical trials conducted on flozins, with a focus on adverse events. AEs—adverse events, UTIs—urinary tract infections, SAEs—serious adverse events.

Drug Name	Conducted Trials	Only Patients with T2DM	Safety with CKD	Safety with Cardiovascular Diseases	Other Drugs Used in Treatment	Observed Adverse Effects
Dapagliflozin	Dapagliflozin monotherapy in type 2 diabetic patients with inadequate glycemic control by diet and exercise [13]	Yes	Not tested	Not tested	Not tested	Most common AEs: nasopharyngitis, diarrhea, and headache—other significant observed AEs: nocturia, increased incidence in signs, symptoms, and other reports suggestive of UTIs and genital infections, hypotensive events, hypoglycemia events
	Efficacy and safety of dapagliflozin monotherapy in people with type 2 diabetes [81]	Yes	Not tested	Not tested	Dapagliflozin/metformin	Most common AE: nasopharyngitis, headache, and influenza—other significant AEs events of hypoglycemia, benign breast neoplasm, events of renal impairment, events suggestive of genital infections and UTIs, pyelonephritis
	Dapagliflozin Versus Glipizide as Add-on Therapy in Patients With Type 2 Diabetes Who Have Inadequate Glycemic Control With Metformin [82]	Yes	Not tested	Not tested	Metformin/dapagliflozin or metformin/glipizide	Most common Aes: nasopharyngitis, hypertension, and influenza—other significant AEs: complex ventricular arrhythmia, decreased calculated creatinine clearance, epigastric pain, prostate cancer, pulmonary embolism, and worsening of coronary artery disease; drug-induced acute hepatitis; hypoglycemic events; renal failure; hypotension; dehydration; hypovolemia; signs and symptoms suggestive of genital infections or UTIs
	Dapagliflozin Is Effective as Add-on Therapy to Sitagliptin With or Without Metformin [83]	Yes	Not tested	Not tested	Dapagliflozin or dapagliflozin/sitagliptin or dapagliflozin/metformin/sitagliptin	Few events of hypoglycemia, one event of major hypoglycemia, symptoms, and events suggestive of genital infection, vulvovaginal mycotic infection, hypotension, dehydration, hypovolemia, renal impairment, prostate neoplasm, thyroid neoplasm
	Dapagliflozin Improves Glycemic Control and Reduces Body Weight as Add-on Therapy to Metformin Plus Sulfonylurea [84]	Yes	Not tested	Not tested	Metformin/sulfonylurea or dapagliflozin/metformin/sulfonylurea	Most common AEs: hypoglycemia, UTIs and genital infections, bronchitis—other significant AEs: two events of renal impairment/failure; orthostatic hypotension; elevated bilirubin
	Long-Term Efficacy of Dapagliflozin in Patients With Type 2 Diabetes Mellitus Receiving High Doses of Insulin [85]	Yes	Not tested	Not tested	Insulin/oral antidiabetic drugs or dapagliflozin/insulin/oral antidiabetic drugs	Most common AEs: nasopharyngitis, UTIs and genital infections, headaches, back pain, hypertension—other significant AEs: hypoglycemia, renal impairment/failure, hypotension, dehydration or hypovolemia
	Safety and Efficacy of Exenatide Once Weekly Plus Dapagliflozin Once Daily Versus Exenatide or Dapagliflozin Alone in Patients With Type 2 Diabetes Inadequately Controlled With Metformin Monotherapy [86]	Yes	Not tested	Not tested	Exenatide/dapagliflozin or exenatide or dapagliflozin	Most common AEs: nausea, UTIs and genital infections, headache, diarrhea—other significant AEs: gastrointestinal AEs, pancreatitis, hypoglycemia
	Dapagliflozin and Cardiovascular Outcomes in Type 2 Diabetes [87]	Yes	Not tested	Yes	Not tested	UTIs and genital infections, symptoms of volume depletion, acute kidney injury, amputations, neoplasms, hepatic events, major hypoglycemic events, diabetic ketoacidosis
	The Dapagliflozin And Prevention of Adverse-outcomes in Heart Failure (DAPA-HF) trial [70]	No	Not tested	Yes	All patients received standard drug and device therapy for heart failure	Only SAEs, AEs leading to treatment discontinuation/interruption/dose reduction, and AEs of special interest were collected: volume depletion, renal dysfunction, major hypoglycemic episodes, fractures, and diabetic ketoacidosis
	Dapagliflozin in Heart Failure with Mildly Reduced or Preserved Ejection Fraction [71]	No	Not tested	Yes	Dapagliflozin, in addition to the patient’s usual therapy	Only SAEs, AEs leading to treatment discontinuation/interruption/dose reduction, and AEs of special interest were collected: UTIs, renal impairment, cardiac failure, COVID-19, pneumonia, ischemic stroke
	Dapagliflozin in Patients with Chronic Kidney Disease [72]	No	Yes	Not tested	Dapagliflozin and ACE inhibitor or ARB for at least 4 weeks before screening	Only SAEs, AEs leading to treatment discontinuation/interruption/dose reduction, and AEs of special interest were collected: amputations, fracture, renal-related AEs, major hypoglycemia, volume depletion
Canagliflozin	Efficacy and safety of canagliflozin monotherapy in subjects with type 2 diabetes mellitus inadequately controlled with diet and exercise [14]	Yes	Not tested	Not tested	Not tested	Selected AEs: UTIs, genital mycotic infection, osmotic diuresis-related AEs, postural dizziness, orthostatic hypotension
	Efficacy and Safety of Canagliflozin Used in Conjunction with Sulfonylurea in Patients with Type 2 Diabetes Mellitus [92]	Yes	Not tested	Not tested	Canagliflozin/sulfonylurea	Selected AEs: UTIs, genital mycotic infections, osmotic diuresis-related events, postural dizziness, orthostatic hypotension, hypoglycemia events
	Efficacy and safety of canagliflozin, an inhibitor of sodium–glucose cotransporter 2, when used in conjunction with insulin therapy in patients with type 2 diabetes [93]	Yes	Not tested	Not tested	Canagliflozin/stable background glucose-lowering therapy	Selected AEs: genital mycotic infections, UTIs, hypoglycemia events, osmotic diuresis-related events, volume-related AEs, renal-related AEs, photosensitivity events, fractures
	Efficacy and safety of titrated canagliflozin in patients with type 2 diabetes mellitus inadequately controlled on metformin and sitagliptin [94]	Yes	Not tested	Not tested	Metformin/sitagliptin or metformin/sitagliptin/canagliflozin	Selected AEs: UTIs, genital mycotic infections, osmotic diuresis-related events, volume depletion-related events, fracture, ketone-related events, hypoglycemia events
	Efficacy and safety of canagliflozin in patients with type 2 diabetes mellitus inadequately controlled with metformin and sulphonylurea [95]	Yes	Not tested	Not tested	Metformin/sulfonylurea or metformin/sulfonylurea/canagliflozin	Selected AEs: UTIs, genital mycotic infection, osmotic diuresis-related AEs, volume-related AEs, hypoglycemia events
	Efficacy and safety of canagliflozin over 52 weeks in patients with type 2 diabetes on background metformin and pioglitazone [96]	Yes	Not tested	Not tested	Metformin/pioglitazone or metformin/pioglitazone/canagliflozin	Selected AEs: UTIs, genital mycotic infection, osmotic diuresis-related events, volume depletion events
	Efficacy and safety of canagliflozin versus glimepiride in patients with type 2 diabetes inadequately controlled with metformin (CANTATA-SU) [97]	Yes	Not tested	Not tested	Canagliflozin/metformin or glimepiride/metformin	Selected AEs: genital mycotic infections, UTIs, osmotic diuresis-related events
	Canagliflozin Compared With Sitagliptin for Patients With Type 2 Diabetes Who Do Not Have Adequate Glycemic Control With Metformin Plus Sulfonylurea [98]	Yes	Not tested	Not tested	Sulfonylurea/metformin/canagliflozin or sulfonylurea/metformin/sitagliptin	Selected AEs: genital mycotic infections, UTIs, pollakiuria, polyuria
	Canagliflozin and renal outcomes in type 2 diabetes: results from the CANVAS Program randomized clinical trials [17]	No	Yes	Not tested	Not tested	Renal AEs: acute kidney injury events, hyperkalemia events
	Kidney, Cardiovascular, and Safety Outcomes of Canagliflozin according to Baseline Albuminuria: A CREDENCE Secondary Analysis [99]	Yes	Yes	Yes	Not tested	Renal AEs: acute kidney injury events, volume depletion events, hyperkalemia events, UTIs, hypoglycemia
Empagliflozin	Empagliflozin monotherapy with sitagliptin as an active comparator in patients with type 2 diabetes [15]	Yes	Not tested	Not tested	Empagliflozin or sitagliptin	Most common AEs: nasopharyngitis, UTIs, hyperglycemia, dyslipidemia—other significant AEs: hypoglycemia, genital infections
	Empagliflozin as Add-On to Metformin in Patients With Type 2 Diabetes [104]	Yes	Not tested	Not tested	Metformin or metformin/empagliflozin	Nasopharyngitis, hyperglycemia, UTIs, genital infections
	Glyxambi (Empagliflozin/Linagliptin): A Dual-Acting Oral Medication Approved for the Treatment of Patients with Type 2 Diabetes [105]	Yes	Not tested	Not tested	Linagliptin or empagliflozin or linagliptin/empagliflozin	Most common AEs: UTIs, genital mycotic infections, upper respiratory tract infections, increased urination, dyslipidemia, arthralgia, nausea—other significant AEs: nasopharyngitis, diarrhea, cough, hypoglycemic events
	Efficacy and safety of the SGLT2 inhibitor empagliflozin versus placebo and the DPP-4 inhibitor linagliptin versus placebo in young people with type 2 diabetes (DINAMO) [106]	Yes	Not tested	Not tested	Empagliflozin or linagliptin	Hypoglycemia
	Initial Combination of Empagliflozin and Linagliptin in Subjects With Type 2 Diabetes [107]	Yes	Not tested	Not tested	Linagliptin/empagliflozin or empagliflozin or linagliptin	Most common AEs: UTIs, upper respiratory tract infections, nasopharyngitis, influenza, hyperglycemia—other significant AEs: hypoglycemic events, volume depletion events, hypersensitivity reactions, pancreatitis
	Comparison of empagliflozin and glimepiride as add-on to metformin in patients with type 2 diabetes [108]	Yes	Not tested	Not tested	Glimepiride/metformin or empagliflozin/metformin	Most common AEs: hyperglycemia, UTIs, nasopharyngitis, upper respiratory tract infections—other significant AEs: genital infections, volume depletion events, bone fracture events
	Initial Combination of Empagliflozin and Metformin in Patients With Type 2 Diabetes [109]	Yes	Not tested	Not tested	Empagliflozin, metformin, or empagliflozin/metformin	Most common AEs: UTIs, upper respiratory tract infections, dyslipidemia, dizziness, diarrhea—other significant AEs: hypoglycemia, genital infections, increased urination events, volume depletion events, dehydration, hypotension
	Impact of empagliflozin added on to basal insulin in type 2 diabetes inadequately controlled on basal insulin [110]	Yes	Not tested	Not tested	Empagliflozin as an add-on to insulin	Most common AEs: hypoglycemia, nasopharyngitis, UTIs, hyperglycemia, dizziness—other significant AEs: genital infections, severe hypoglycemic events
	Empagliflozin and Cardiovascular and Kidney Outcomes across KDIGO Risk Categories [18]	Yes	Yes	Yes	Empagliflozin as an add-on to the standard of care for T2DM and cardiovascular risk management	Most common AEs: UTIs, genital infections—other significant AEs: bone fracture, hyperkalemia, hypoglycemia
	Empagliflozin in Patients with Chronic Kidney Disease [19]	No	Yes	Yes	Empagliflozin as an add-on to single-agent RAS inhibitor	Selected AEs: UTIs, genital infections, hyperkalemia, acute kidney injury, dehydration, liver injury, ketoacidosis, lower limb amputation, bone fracture, hypoglycemia
	Efficacy and safety of empagliflozin added to existing antidiabetes treatment in patients with type 2 diabetes and chronic kidney disease [20]	Yes	Yes	Not tested	Empagliflozin as an add-on to antidiabetic drugs (excluding other SGLT2 inhibitors)	Selected AEs: hypoglycemia, UTIs, upper respiratory tract infections, nasopharyngitis, hyperglycemia, back pain, nausea, volume depletion
	Cardiovascular and Renal Outcomes of Empagliflozin in Heart Failure [22]	No	Not tested	Yes	Empagliflozin as an add-on to treatment for heart failure (diuretics, inhibitors of the renin–angiotensin system, and neprilysin, beta-blockers, mineralocorticoid receptor antagonists, and cardiac devices)	Selected AEs: hypotension, volume depletion, hypoglycemia, UTIs, genital infections, bone fractures, lower limb amputation
Ertugliflozin	Phase III, efficacy and safety study of ertugliflozin monotherapy in people with type 2 diabetes mellitus inadequately controlled with diet and exercise alone [16]	Yes	Not tested	Not tested	Not tested	Selected AEs: UTIs, genital mycotic infections, hypoglycemia, hypovolemia, pollakiuria, polyuria, constipation
	Long-term efficacy and safety of ertugliflozin monotherapy in patients with inadequately controlled T2DM despite diet and exercise [117]	Yes	Not tested	Not tested	Metformin in phase B of the study in participants from the placebo group	Selected AEs: genital mycotic infections, UTIs, hypoglycemia, hypovolemia, pollakiuria, polyuria, one event of hepatic adjudication
	Effect of ertugliflozin on glucose control, body weight, blood pressure, and bone density in type 2 diabetes mellitus inadequately controlled on metformin monotherapy (VERTIS MET) [118]	Yes	Not tested	Not tested	Ertugliflozin as an add-on to metformin	Selected AEs: genital mycotic infections, UTIs, hypoglycemia, hypovolemia, pollakiuria, dizziness, orthostatic blood pressure decrease events
	Ertugliflozin Compared with Glimepiride in Patients with Type 2 Diabetes Mellitus Inadequately Controlled on Metformin (VERTIS SU) [119]	Yes	Not tested	Not tested	Ertugliflozin or glimepiride	Selected AEs: hypoglycemia, genital mycotic infections, UTIs, hypovolemia, acute kidney injury event, diabetic ketoacidosis event, toe amputation events
	Ertugliflozin plus sitagliptin versus either individual agent over 52 weeks in patients with type 2 diabetes mellitus inadequately controlled with metformin (VERTIS FACTORIAL) [120]	Yes	Not tested	Not tested	Ertugliflozin or sitagliptin	Selected AEs: genital mycotic infections, UTIs, hypoglycemia, hypovolemia, diabetic ketoacidosis event
	Ertugliflozin and Sitagliptin Co-initiation in Patients with Type 2 Diabetes (VERTIS SITA) [121]	Yes	Not tested	Not tested	Ertugliflozin as an add-on to sitagliptin	Selected AEs: genital mycotic infections, UTIs, hypoglycemia, hypovolemia, a decrease from baseline of >30% in eGFR events
	Efficacy and safety of the addition of ertugliflozin in patients with type 2 diabetes mellitus inadequately controlled with metformin and sitagliptin (VERTIS SITA2) [122]	Yes	Not tested	Not tested	Ertugliflozin as an add-on to sitagliptin and metformin	Selected AEs: genital mycotic infections, UTIs, hypoglycemia, hypovolemia
	Efficacy of Ertugliflozin on Heart Failure–Related Events in Patients With Type 2 Diabetes Mellitus and Established Atherosclerotic Cardiovascular Disease [23]	Yes	Not tested	Yes	Not tested	---
	Ertugliflozin in Patients with Stage 3 Chronic Kidney Disease and Type 2 Diabetes Mellitus (VERTIS RENAL) [21]	Yes	Yes	Not tested	Not tested	Selected AEs: UTIs, genital mycotic infections, hypoglycemia, hypovolemia, albuminuria events

## Data Availability

This review summarizes data reported in the literature and it does not report primary data.
